# Supplementary material for: Knowledge, attitude, practices, and determinants of them toward tuberculosis among social media users in Bangladesh: A cross-sectional study
Source: PLoS One. 2022 Oct 11;17(10):e0275344. doi: 10.1371/journal.pone.0275344 (PMC9553051; doi:10.1371/journal.pone.0275344)
Supplement: S1 Questionnaire — (DOCX) [file pone.0275344.s001.docx]

Knowledge, Attitude, Practices, and Determinants of Them towards Tuberculosis in Bangladesh: A Cross-sectional Study

**Consent**

Assalamu Alaikum/Adab (Greetings). I would like to request you to take part in a small survey. This study aims to assess the knowledge, attitude, and practices toward TB in Bangladesh. The survey includes questions regarding your knowledge, attitude, practices towards TB, Risk behaviors related to TB, and some socio-demographic questions. I hope this information will help the Bangladesh government and policymakers to make a better strategy to mitigate the burden of TB. You will not be asked any personal or sensitive questions, nor will you be asked your name/identity. The survey may take 4-5 minutes to complete. Please note that your participation in the survey is entirely voluntary. You can reject it anytime during the survey and skip any questions if they seem sensitive. The results will be reported in aggregate form and will be used only for research purposes.

Are you willing to participate in this survey?

1. Yes
2. No

If the answer is ”NO”, it shows “Thank you for your time” and ends the survey. Otherwise, the survey continues.

**Section 1: Background characteristics of the respondents**

| **Sl#** | **Questions** | **Types of code** | | **Direction** | **Comments** |
| --- | --- | --- | --- | --- | --- |
| 101. | Gender | Male  Female  Others | 1  2  3 |  |  |
| 102. | How old are you? | 15-29  30-44  45-59  60-74  75+ | 1  2  3  4  5 |  |  |
| 103. | What is your current marital status? | Married  Unmarried  Divorced  Widowed  Separated | 1  2  3  4  5 |  |  |
| 104. | How many years of education have you completed till now? | Less or equal SSC  HSC  Undergraduate  Master or higher  Never been to school | 1  2  3  4  99 |  |  |
| 105. | What was your total income in the last month? | ≤10 thousand  11-20 thousand  21-30 thousand  31-40 thousand  ≥40 thousand  No income | 1  2  3  4  5  99 |  |  |
| 106. | Occupation | Business  Housewife  Govt. employee  Non-govt. employee  Unemployed  Self-employed  Student  Others .............................. | 1  2  3  4  5  6  7  97 |  |  |
| 107 | Religious | Muslims,  Hindu,  Buddhists/Cristian  Other | 1  2  3  97 |  |  |
|  | Where do you live? | Urban  Rural | 1  2 |  |  |
|  | | | | | |

| **Sl#** | **Questions** | **Types of code** | | **Direction** | **Comments** |
| --- | --- | --- | --- | --- | --- |
| 201. | Do you have diabetes? | Yes  (Did investigation)  No  (Did investigation)  Never investigate  Tested but didn’t collect investigation report  Don’t know  No responses | 1  2  3  4  97  98 |  |  |
| 202. | Do you smoke (biri/ cigarette) regularly? | Yes  Never | 1  2 |  |  |
| 203. | If Q. 302 is yes, how many cigarettes per day (on average)? | # cigarettes per day | ------- |  |  |
| 204. | Did you drink alcohol in the last three months? | Yes  No  Don’t know  No responses | 1  2  97  98 |  |  |
| 205. | Have you been exposed to indoor cooking smoke? | Yes  No | 1  2 |  |  |

**Section 2:** **Risk behaviours** **related to TB**

**Section 3: Knowledge of Tuberculosis**

(Do not probe. Now I will discuss some questions on knowledge of TB)

| **Sl#** | **Questions** | **Types of code** | | **Direction** | | **Comments** |
| --- | --- | --- | --- | --- | --- | --- |
| **Source of knowledge on TB** | | | | | | |
| 301. | Did you hear anything about TB? | Yes  No  Don’t know | 1  2  97 |  | |  |
| 302 | From where and whom did you learn about TB?  (Multiple answers acceptable) | Exposure to TB treatment  Inmates suffering from TB  TV/Radio/Newspaper  Leaflets/Poster/Signboard/Billboard  Friend/Relatives/Family Member  Health professionals Religious leaders/teacher  Internet  Others (specify) | 1 0  1 0  1 0  1 0  1 0  1 0  1 0  1 0  1 0 |  | |  |
| **Knowledge about TB causes** | | | | | | |
| 303 | What is the primary cause of TB?  (Only one response) | TB germ /Bacteria  Virus  Cold wind  Smoking  Spoiled soil (soil with a bad odor)  Poor hygiene Alcohol  Inherited  Don’t know | 1  2  3  4  5  6  7  97 | 1 | |  |
| **Knowledge about the transmission of TB** | | | | | | |
| 304 | TB is spread from person to person through the  air when coughing or sneezing? | Yes  No  Don’t know | 1  0  99 | 1 | |  |
| 305 | TB can be transmitted by sharing utensils? | Yes  No  Don’t know | 1  0  99 | 0 | |  |
| 306 | TB can be transmitted through food? | Yes  No  Don’t know | 1  0  99 | 0 | |  |
| 307 | TB can be transmitted through sexual contact? | Yes  No  Don’t know | 1  0  99 | 0 | |  |
| 308 | What is the most common site for TB infection in the body? (Only one answer) | Lungs  Glands  Brain  Bones  Others (specify) ---  Don’t know | 1  2  3  4  5  99 | 1 | |  |
| **Knowledge about symptoms of TB** | | | | | | |
| 309 | A person who is infected with TB coughs for several (more than 3)  weeks? | Yes  No  Don’t know | 1  0  99 | 1 | |  |
| 310 | A person who is infected with TB has persistent  fever? | Yes  No  Don’t know | 1  0  99 | 1 | |  |
| 311 | A person who is infected with TB sweats during  the night? | Yes  No  Don’t know | 1  0  99 | 1 | |  |
| 312 | A person who is infected with TB has pain in the  chest or back? | Yes  No  Don’t know | 1  0  99 | 1 | |  |
| 313 | Is weight loss one of the symptoms of TB? | Yes  No  Don’t know | 1  0  99 | 1 | |  |
| **Knowledge about availability of TB treatment** | | | | | | |
| 314. | Is TB management available free of cost in Bangladesh? | Yes  No  Don’t know | 1  0  99 | | 1 |  |
| 315. | Is TB curable? | Yes  No  Don’t know | 1  0  99 | 1 | |  |

| **Attitudes and practices about TB** | | | | | |
| --- | --- | --- | --- | --- | --- |
| 401 | In your opinion, how serious disease is TB?  (chose only one) | Very serious  Somewhat serious  Not very serious | 1  2  99 | 1 |  |
| 402 | Are you afraid of getting infected with TB? (chose only one) | Yes  No  Don’t know | 1  0  99 | 1 |  |
| 403 | Will you keep it secret when any family member gets TB? | Yes  No  Don’t know | 1  0  99 | 0 |  |
| 404 | Would you be willing to work with someone previously treated for TB? | Yes  No  Don’t know | 1  0  99 | 1 |  |
| 405 | What would be your reaction if you were found out that you have TB? (chose only one) | Visit health facility  Fear  Shame  Sadness/hopelessness  Others (specify) | 1  2  3  4  5 | 1 |  |
| **Practices** | | | | | |
| 501 | If you had symptoms of TB, at what point would you go to the health facility? (chose only one) | When treatment on my own does not work.  When symptoms that look like TB last for 3–4 weeks.  As soon as I realize that my symptoms might be related to TB.  I would not go to the doctor. | 1  2  3  4 | 3 |  |
| 502 | If you had symptoms of TB, where would you go for TB treatment? (chose only one) | Modern drugs  Herbal Remedies  Home Remedies  Praying /holy water  Don’t Know | 1  2  3  4  5 | 1 |  |
|  |  |  |  |  |  |
