# Supplementary material for: Knowledge, attitude, practices, and determinants of them toward tuberculosis among social media users in Bangladesh: A cross-sectional study
Source: PLoS One. 2022 Oct 11;17(10):e0275344. doi: 10.1371/journal.pone.0275344 (PMC9553051; doi:10.1371/journal.pone.0275344)
Supplement: Attachment — Submitted filename: Response to reviewers_R2.docx [file pone.0275344.s003.docx]

**Responses to reviewers:**

**Reviewer point#1:**  *Section 2.1 line 6 mentions that participants "were requested to help those who do not have access to the internet or social media". What kind of help was suggested? If these "others" had no access to internet, that would imply that devices with internet access had to be shared. However, in section 2.5 it is mentioned that duplicate submissions were identified and dropped based on the device ids.*

**Author response#1:** Thank you for identifying this significant discrepancy. Actually, our initial plan was to request participants to help others who don’t have internet/smart device access. However, later, we decided to survey only those who have internet/social media access. The sentence was taken from our initial study plan doc, and regrettably, we kept this sentence by mistake. The statement in section 2.5 about removing duplicate submissions based on device id is correct.

**Reviewer point#2:**  *There are still some grammatical and typographical errors. Eg. device is spelled devise in section 2.5.*

**Author response#2:** We are sorry for the grammatical and typographical errors. We have gone through the entire manuscript with extensive care and corrected all the errors we identified.

**Reviewer point#3:**  *References: Some references are not in the correct format. Please check the correct format for reports and websites*

**Author response#3:** Thank you so much for identifying the discrepancies in the references. We have corrected the discrepancies in the revised manuscript.

**Reviewer additional point:**  *Rather than attempt to extrapolate the results to the general population, the authors can focus on the interpretation of these results among social media users and their implications. The discussion can be rewritten with focus on the interpretation of this study's results and how they can inform future targeted health education using social media.*

**Author response:** Thank you for your insightful comment, we really appreciate it. We have rewritten the discussion section following your instructions.
