# Supplementary material for: Knowledge, attitude, practices, and determinants of them toward tuberculosis among social media users in Bangladesh: A cross-sectional study
Source: PLoS One. 2022 Oct 11;17(10):e0275344. doi: 10.1371/journal.pone.0275344 (PMC9553051; doi:10.1371/journal.pone.0275344)
Supplement: Attachment — Submitted filename: Response to Reviewers_R3.docx [file pone.0275344.s004.docx]

**Responses to reviewers:**

***Reviewer point#1:*** *The authors have made all suggested changes. I have a few suggestions.
The title may be better if including the term internet users or social media users.*

**Author response#1:** Thank you for your suggestion. The title now includes social media users to specify the study participants.

**Reviewer point#2:**  *In socio-demographic characteristics(Section3.1)- is there a need to include calculated precision?*

**Author response#2:** Yes, you are absolutely right. We have removed it.

**Reviewer point#3:**  In Table 1 - no need to include Consent as a variable.

**Author response#3:** This variable from Table 1 has been removed.

**Reviewer point #4:**  The article can be further shortened, figures 2,3,4 are not necessary. Similarly, the data in table 2 can be included in tables 4,5,6 instead of a separate table.

**Author response #4:** The article has been shortened significantly following the above suggestions. Thank you for the suggestions.

**Reviewer point#5:** In the discussion section comparing the demographic characteristics of the general population with that of this study may be useful. The Discussion can be streamlined further. In the Discussion Paragraph 5 on Pg14 states TB is significantly prevalent among the young population in Bangladesh who are mainly active users of social media. This statement is abrupt and requires either reference or further explanation. Or this statement can be included in the first paragraph where the importance of the 18-45 years age group is mentioned.

**Author response #5:** We agree with you that the sentence was abrupt without any references. We have revised the wording to make it coherent and also added a reference. We think the discussion section is good overall.

**Reviewer point#6:** Please check format for references for reports and databases. Reports as references year of publication has to be mentioned (eg. Ref no. 24). For databases references should be according to ICJME guidelines(e.g. Ref: 22).

**Author response #6:** Thank you for identifying the issue and also for sharing the guidelines. We have modified them following the guidelines.
